# Antigen recognition in autoimmune diabetes: a novel pathway underlying disease initiation

**DOI:** 10.1093/pcmedi/pby015

**Published:** 2018-11-23

**Authors:** Xiaoxiao Wan, Emil R Unanue

**Affiliations:** Department of Pathology and Immunology, Washington University School of Medicine, St. Louis, MO, USA

## Abstract

Development of human autoimmune disorders results from complex interplay among genetic, environmental, and immunological risk factors. Despite much heterogeneity in environmental triggers, the leading genes that give the propensity for tissue-specific autoimmune diseases, such as type 1 diabetes, are those associated with particular class II major histocompatibility complex alleles. Such genetic predisposition precipitates presentation of tissue antigens to MHC-II-restricted CD4 T cells. When properly activated, these self-reactive CD4 T cells migrate to the target tissue and trigger the initial immune attack. Using the non-obese diabetic mouse model of spontaneous autoimmune diabetes, much insight has been gained in understanding how presentation of physiological levels of self-antigens translates into pathological outcomes. In this review, we summarize recent advances illustrating the features of the antigen presenting cells, the sites of the antigen recognition, and the nature of the consequent T cell responses. We emphasize emerging evidence that highlights the importance of systemic presentation of catabolized tissue antigens in mobilization of pathogenic T cells. The implication of these studies in therapeutic perspectives is also discussed.

## Introduction

Type 1 diabetes (T1D) is a prototypic tissue-specific autoimmune disease characterized by lymphocytic infiltration into the islets of Langerhans, resulting in loss of insulin-producing β cells and hyperglycemia. Genome-wide association studies have identified that the primary T1D-susceptibility loci are the Class II human leukocyte antigen (HLA) genes in the major histocompatibility complex (MHC).^1,2^ Class II MHC (MHC-II) molecules with a strong link to a high disease risk, such as HLA-DQ8 and HLA-DQ2, display a unique structural property, that is, substitution of the aspartic acid at the 57th position of the MHC-II β chain.^3–6^ Consequently, these MHC-II molecules select a much distinct MHC-II peptidome that largely favors binding of peptides having acidic residues at the p9 position.^7–9^ Therefore, islet-derived antigens containing such binding motifs, including insulin^5^ and glutamic decarboxylase acid (GAD),^10^ can be presented, generating a CD4 T cell repertoire skewing to diabetogenic reactivity. Although the course of the disease can be modified by various environmental factors,^11^ these fundamental findings demonstrate a tight link between genetic susceptibility and antigen presentation, which in concert results in an autoimmune response.

Development of T1D in the non-obese diabetic (NOD) mouse requires expression of the I-A^g7^ MHC-II molecule.^12,13^ Importantly, I-A^g7^ is structurally homologous to the human HLA-DQ8 and HLA-DQ2; it also lacks the aspartic acid at the β chain 57th position. Thus, both I-A^g7^ and HLA-DQ8 select similar peptidomes,^9^ including diabetogenic antigens. Transgenic modification^14^ of I-A^g7^ or expression of a different MHC haplotype (I-A^b^)^15^ abolished T1D development in NOD mice. Several key immunological features found in human T1D are recapitulated in the NOD mouse: 1) early appearance of anti-islet antibodies in the serum with predictive values of disease onset^16^; 2) local invasion of CD4 and CD8 T cells recognizing β cell antigens,^17^ forming a characteristic lesion in the islets, termed insulitis; 3) chronic disease progression as a result of the effects of regulatory mechanisms to counter the pathogenic elements^18^; and 4) persistence of autoreactive T cell responses post β cell destruction,^19^ leading to rapid rejection of islet transplants into already diabetic individuals.

More importantly, the NOD mouse is among few models that spontaneously develop an autoimmune disease without the need for immunization with a putative antigen. This valuable trait allows for examining antigen presentation events that naturally take place to initiate autoreactivity, a serious clinical problem difficult to study in human patients. This issue can be further extrapolated to several questions: 1) What are the major antigens required for disease initiation? 2) What are the features of the antigen presenting cells (APCs)? 3) Where are the antigens being presented? 4) How can a tissue antigen be made available to T cells? 5) How does self-recognition generate pathogenic responses? To place these questions in perspective, we first briefly summarize studies that have examined insulin epitopes and the clonotypic T cell reactivity. This topic was discussed in detail in our recent reviews.^20,21^

## Insulin epitopes and autoreactivity

It is conceivable that diverse antigens are involved during progression of a complex autoimmune disease; the term “epitope-spreading” has been used in this regard. However, at the initial phase, only a limited number of antigens may be required to spark the process. This is an attractive hypothesis because such antigens could be targeted by antigen-specific therapies to achieve disease prevention. Indeed, insulin has been identified as the prime antigen essential for T1D initiation in the NOD mouse. The initial evidence is summarized as follows^16,21–29^: 1) Insulin autoantibodies (IAAs) are detected long before disease onset in NOD mice and in patients with T1D, and therefore serve as a biomarker for disease prediction; 2) According to genome-wide association studies, polymorphism in the promotor region of the insulin locus is the second strongest risk-conferring factor in T1D, following HLA; 3) Genetic overexpression of insulin in APCs reduces T1D incidence in NOD mice; 4) Insulin-reactive T cells transfer diabetes in non-diabetic hosts; and 5) Genetic ablation of insulin expression in the thymus accelerates T1D development in NOD mice.

The definitive evidence was obtained using NOD mice containing a transgene coding a mutant insulin B chain with a tyrosine to alanine change at the 16th position (referred to as B16A).^30^ This mutation maintained bioactivity of the insulin molecule; however, development of IAAs, insulitis, and clinical diabetes was completely suppressed.^30^ By contrast, the T cells in the B16A mice were able to reject normal islets expressing wild-type insulin.^31^ Therefore, without recognition of a small fragment of the insulin B chain, reactivity to other antigens cannot elicit effective pathogenic responses. These studies suggest that tissue-specific autoimmunity is not driven by random self-antigens but is finely programmed by recognition of highly selective peptide-MHC complexes (pMHCs), which elicits downstream epitope-spreading. To note, insulin-specific T cells, including those reactive to B:9–23, have been identified in the islets of T1D patients.^32,33^

Using synthetic peptides and T cells as a biological probe, a series of fundamental studies have demonstrated that the “hot spot” of the insulin B chain that generates immunogenicity is the 9–23 segment (B:9–23; SHLVEALYLVCGERG).^30,31,34–38^ We have subsequently determined that this peptide contained two 9-mer I-A^g7^-binding registers, B:12–20 (VEALYLVCG) and B:13–21 (EALYLVCGE), recognized by two specific sets of CD4 T cells.^38,39^ Although these two epitopes differ by only a one amino acid shift, the mechanisms underlying their processing and presentation are profoundly distinct. B:13–21 has a higher binding affinity to I-A^g7^ because of an acidic residue (Glu) at p9, and is therefore selectively presented when the conformational insulin protein is internalized and processed by APCs.^39,40^ Epitopes with weaker affinities, including B:12–20, are released by the peptide editor H2-DM in the late endosomal compartments; however, B:12–20 can be presented when free insulin peptides or denatured insulin are offered to APCs via direct loading onto MHC-II molecules at the cell surface or in the early endosomes, where H2-DM is absent.^39,40^

We have identified CD4 T cells that recognize B:13–21 or B:12–20 and refer to them as type-A and type-B T cells, respectively. The two sets of T cells display distinct features in their specificity, selection, and effector function. Because insulin expression by medullary thymic epithelial cells (mTECs) is known to mediate negative selection of autoreactive clones, it is possible that the B:13-21-specific T cells are deleted or functionally silenced in the thymus, and those specific to B:12–20 may escape. Indeed, most of the insulin-reactive T cell clones found in the periphery reacted only to insulin peptides not to the protein.^38,39^ Functional assays also confirmed that B:12-20-reactive T cells were able to mount a robust response upon antigen stimulation, whereas those specific to B:13–21 showed little reactivity.^39^ Importantly, such defect was not found in B16A mice.^39^ These results were further supported by the 8F10 T cell receptor transgenic mouse to B:12–20, in which the 8F10 T cells escaped thymic selection, seeded the periphery, caused diabetes,^41^ and were able to facilitate germinal center formation for production of class-switched IAAs.^42^ The importance of T cells with a “type-B” feature has been highlighted in other autoimmune diseases in mice and humans.^43–48^ A notable example is a recent study that characterized hypocretin-specific CD4 T cells in patients with narcolepsy; most of these T cells recognized only the exogenous peptides not those post protein-processing.^49^

## Antigen presentation in the islet: the resident macrophages

Defining the role of insulin peptides and the type-B T cells in T1D provides a platform to answer a central question: How do the β cells communicate with the CD4 T cells? β cells do not express MHC-II, which suggests that there is an intermediate cell population in the islet that can transform β cell-derived products into the correct pMHCs for recognition by CD4 T cells.

We examined the myeloid cell compartment in purified islets and found that the islet harbored a population of tissue-resident macrophages exhibiting unique features. The islet contained a small portion (1–5%) of hematopoietic cells (defined by CD45^+^); this finding holds true in all the mouse strains examined.^50^ In C57BL/6 (B6) mice, ~90–95% of the hematopoietic cells were of macrophage lineage (defined by CD45^+^CD11c^+^F4/80^+^CD11b^+^)^50^; in 3-week-old NOD female mice, the macrophages were also the dominant component (~85–90%), with the rest being a few dendritic cells (DCs) and infiltrating T cells.^51^ Of note, the islet macrophages originated from definitive hematopoiesis and could not be replaced by blood monocytes at steady state.^50^

The islet macrophages were also transcriptionally active; high levels of expression of genes encoding MHC-II and costimulatory molecules^52^ support a role as competent APCs. High levels of expression were also found in a wide range of inflammatory genes, including chemokines, chemokine receptors, toll-like receptors, and cytokines.^52^ In general, these genes represented active NFκb signaling,^52^ a feature consistent with other barrier-tissue macrophages.^53,54^ At the protein level, we confirmed that these macrophages spontaneously produced TNFα and pro-IL-1β.^52^ The islet macrophages are intrinsically active regardless of autoimmunity in that the basal level of activation was found in the transcriptomes of both NOD mice and the B6 mice expressing the I-A^g7^ haplotype (B6g7), a strain that does not develop T1D.^52^ Although the homeostatic function of islet macrophages during adulthood is not completely clear, this poised activation phenotype may be a self-defensive mechanism that protects the islet from blood pathogens. Indeed, the islet macrophages rapidly responded to low concentrations of circulating LPS and were able to capture microparticles from the vasculature.^55^ This was partially supported by two-photon imaging analysis showing that the islet macrophages were anchored near the blood vessels and constantly probed the lumen via extending filopodia, an activity finely controlled by local glucose concentrations.^55^ The islet macrophages also expressed purinergic receptors, and a recent study proposed that they can sense ATP released in the islet, a means to monitor β cell activity.^56^

Ultrastructural analysis by electron microscopy revealed that the islet macrophages were in intimate contact with the β cells.^55^ Importantly, they acquired intact insulin dense core granules (DCGs), a process requiring cell contact and mobilization of intracellular calcium.^55,57^ Consequently, the islet macrophages were spontaneously loaded with insulin products and could activate the cognate T cells without the need to pulse with exogenous antigens. Early CD4 T cells entering the islet were found to be in close contact with the macrophages.^58^ These findings raised an important question: What is the role of the islet macrophages in T1D? Using a monoclonal antibody (AFS98) that preferentially depleted the islet macrophages through blockade of CSF-1R signaling, we found that T1D development in NOD mice was largely abrogated.^51^ It seemed that treatment with AFS98 established a “local tolerance” state, in which the islet was specifically protected from immune infiltration, whereas sensitization of diabetogenic T cells in the periphery was unaffected.^51^ The pathogenic function of the islet macrophage was shown to operate widely across disease course, with disease protection observed when AFS98 administration was started in either 3- or 10-week-old female NOD mice.^51^

## Origin of insulin epitopes

The major component of regular DCGs is the insulin molecule (~300 000 molecules/granule). The passage of these granules to the islet macrophages should lead to presentation of the B:13–21 epitope. However, surprisingly, the islet macrophages preferentially activated the B:12-20-specific type-B T cells.^57^ This suggests that free insulin peptides and/or denatured insulin are the source of the B:12–20 epitope and may explain how the type-B T cells found proper conditions for their activation in the target tissue.

To locate the insulin peptides, we developed two monoclonal antibodies, one that specifically recognized B:9–23 (clone AIP),^38,57,59^ and another that recognized the entire insulin B chain (B:1–30; clone 6F3.B8)^59^; neither antibody reacted to native insulin. Immunofluorescent analysis revealed a distinct positioning pattern of B:9–23 and B:1–30 in the islets; B:9–23 did not co-stain with insulin but colocalized with LAMP-1,^38^ a marker of lysosomes, whereas B:1–30 largely co-stained with insulin and was found in nearly all the β cells.^59^ These findings were supported by further characterization of two sets of β cell vesicles that differed in their biology and contents. We determined that the B:9-23-containing vesicles are compatible with the crinophagic bodies described in β cells as well as other endocrine organs.^60,61^ These vesicles are generated through fusion of the regular DCGs with lysosomes, by which the excessive number of insulin DCGs are removed to maintain cellular homeostasis.^62^ After lysosomal degradation, a series of catabolized peptides are generated which can be identified in the crinophagic bodies isolated by differential centrifugation.^57,59^ On the other hand, using immunogold conjugated 6F3.B8 and electron microscopy, we confirmed the presence of the intact B chain together with insulin molecules in regular DCGs.^59^ We speculate that the intact B chain segments are generated via natural denaturation of the interchain disulfide bonds of the highly packed insulin molecules.

We emphasize that generation of catabolized insulin fragments via the crinophagic pathway is a physiological phenomenon regardless of prior inflammation; this occurs naturally in both B6 and NOD mice as well as in non-diabetic humans.^59^ Using mass spectrometry analysis, we compared the peptidome between the crinophagic bodies and the insulin DCGs.^59^ In three different mouse strains and humans, the insulin DCGs mostly contained C peptides and the long B chain. By contrast, the crinophagic bodies were rich in a diversity of short peptides. In particular, peptides associated with the 9–23 region of the B chain, including B:9–23 and B:11–23, were exclusively found in the crinophagic bodies.

Three findings attracted our attention: first, many peptides found in the crinophagic bodies contained pathogenic epitopes compatible with those predicted in previous studies using autoreactive T cell clones (discussed in the next section). Second, many of the peptides with potential immunogenicity were natural sequences. As pointed out in a recent review,^63^ although neoantigens can be generated by various post-translational modification mechanisms, their contribution to spontaneous T1D development requires further exploration. Third, the segregation patterns of the insulin peptides in the two sets of vesicles are very similar between mouse and non-T1D human islets. Immunogenicity may be elicited in individuals who carry MHC molecules able to bind these peptides. We identified a sequence representing insulin B:11–30 in human crinophagic bodies; this long peptide contained the B:11–23 epitope recognized by T cells in T1D subjects expressing HLA-DQ8.^64^

## Sensitizing the periphery: peptide release

There is ample evidence showing that low concentrations of circulating insulin can be presented to cognate T and B cells in lymphoid tissues. In brief (see detailed reviews in^20^), insulin presentation influenced development of T and B cells in the thymus and in the bone marrow, respectively. In secondary lymphoid tissues, insulin presentation played a role in functional silencing of autoreactive B cell clones. In a recent study, we found that 8F10 T cells differentiated into competent T follicular helper cells, facilitating formation of insulin-specific germinal centers.^42^ The cognate T-B cell interactions took place in various lymphoid organs, including the spleen and different lymph nodes.^42^ There are two implications of these results: 1) peripheral insulin presentation was not restricted in the pancreatic draining lymph node (pLN) but was disseminated throughout the lymphoid structures; and 2) the B:12–20 epitope was presented to the 8F10 T cells in the periphery, indicating availability of free insulin peptides.

Systemic presentation of insulin epitopes was confirmed in a physiological setting.^59^ Using two-photon imaging analysis, we found that naïve 8F10 T cells, when adoptively transferred into young NOD recipients, had reduced velocities in various lymph nodes relative to co-transferred polyclonal CD4 T cells. Because T cells constrained their motility during antigen-specific interactions, this finding was proof of peripheral insulin recognition. Subsequent analyses reached several important conclusions pertinent to this issue.^59^ First, insulin recognition by 8F10 T cells was precisely guided by the specific epitope, as these T cells maintained normal motility in the B16A mouse. Second, such recognition required expression of I-A^g7^ but not the NOD background; it occurred in diabetes-resistant B6 mice expressing the I-A^g7^ haplotype. Third, both conformational molecules and free peptides were sources of the insulin epitopes. This was determined by *in vivo* blockade of the insulin receptor, which inhibited uptake of the insulin molecule and presentation by APCs. Under such settings, motility arrest of the 8F10 T cells was unaffected.

The insulin peptides that sensitized the lymph nodes were released from the islets.^59^ In standard insulin secretion assays, insulin peptides, including B:9–23 and B:1–30, were secreted from the islets along with insulin molecules upon glucose challenge. Using mass spectrometry to map the secreted peptides, we identified many sequences that were also found in the two sets of β cell granules. For example, peptides in the crinophagic bodies, especially those related to B:9–23, were identified as a component of the released peptidome. It is worth noting that secreted insulin B chain peptides constituted only a small portion of all the insulin peptides, among which the C-peptide was most abundant. Considering the essential role of B:9–23 in driving T1D, these results support the notion that the major autoreactivity comes from recognition of a minor component of self-antigens.

Notably, these peptides contained most of, if not all, the immunogenic epitopes predicted in previous studies. We found a panel of peptides spanning the junction of the B chain and the C peptide (B-C spanning). A peptide with similar composition was shown to be involved in T1D pathogenesis as an early autoantigen.^65^ In addition to MHC-II epitopes, such as B:12–20 and B:13–21, we found the pathogenic B:15–23 MHC-I (H2-K^d^) epitope recognized by the G9C8 CD8 T cells.^66^ Moreover, three peptides of the A chain contained the A:14–20 epitope, which bound to H2-D^b^ despite lacking a C-terminal anchor residue and activated highly pathogenic AI4 CD8 T cells.^67,68^ The presence of the MHC-I epitopes is pertinent to an enlightening study that identified circulating CD8 T cells in both healthy and diabetic subjects responding to HLA-I peptides derived from β cells.^69^ Importantly, many HLA-I-bound peptides in β cells were derived from proteins associated with secretory granules,^69^ raising the possibility that they may be generated via degradation in the crinophagic bodies. Whether and how the crinophagic pathway plays a role in delivering the peptides to the MHC-I presentation machinery remains an important issue to be addressed.

## Impacts on T cell biology during peripheral antigen recognition

Further evidence that insulin peptides entered the circulation came from experiments that identified a sequence representing B:9–23 in mouse urine.^58^ Seeding of insulin peptides into the periphery led us to determine whether this process influenced T cell biology, an important issue concerning the essential role of the entire peripheral lymphoid system for development of full-blown diabetes. Specifically, surgical removal of the pLN in 3-week-old NOD mice led to a reduction in diabetes in ~25% of the control mice,^70^ implying the availability of bioactive T cells in such mice. By contrast, ablation of all lymph nodes by administration of lymphotoxin-β receptor-Ig fusion protein to the pregnant mice completely abolished diabetes development and the reactivity of diabetogenic T cells.^71^ These two findings highlight the importance of other peripheral lymph nodes, in addition to the pLN, in interactions with T cells.

We compared the transcriptional profile of the 8F10 T cells transferred into NOD or B16A mice. In the former the T cells are exposed to insulin peptides but not in the latter. We found that recognition of insulin peptides had imposed an effector-like signature in the 8F10 T cells.^58^ Specifically, during antigen recognition, the 8F10 T cells turned on a transcriptional program that reflected T cell activation and effector function, but not T cell anergy, tolerance, and exhaustion.^58^ In support of these findings, 8F10 T cells with prior exposure to insulin peptides responded more robustly upon a secondary antigen challenge *in vitro*; they underwent a greater degree of cell division and produced more effector cytokines.^58^ More importantly, they accelerated diabetes development when transferred into lymphopenic recipients.^58^

Considering the nature of the B:12–20 epitope, these findings were unexpected. The low affinity of this epitope with I-A^g7^ has been demonstrated previously, indicating a fast dissociation rate.^39^ It is also expected that the amount of the pMHCs displayed by peripheral APCs is very low. The low affinity/avidity of the B:12–20 presentation may not cause a full program of T cell activation. This is evident by the finding that the 8F10 T cells only underwent significant proliferation when they migrated into the islets, where the antigens were considerably more abundant, but not when they migrated to peripheral sites, including the pLN.^41^ However, repeated encounters of the B:12–20 epitope had positive biological effects in the T cells, an issue relevant to previous studies showing that T cells can accumulate and integrate sequential signals upon antigen encounters to reach a threshold triggering activation.^72,73^ Furthermore, the result that prior antigen exposure enhanced 8F10 T cell pathogenicity is reminiscent of elegant studies showing that tonic self-recognition promoted efficient T cell responses to foreign antigens.^74^ At the molecular level, the transcriptional profile of 8F10 T cells undergoing continuous antigen recognition correlated with an effector-like phenotype but not exhaustion, as it happens on some chronic viral infections. This difference may be attributed to a much higher density of effective viral-specific pMHCs during viral infection.^75^ Although the example of the 8F10 T cell highlights that diabetogenic potential can be influenced in peripheral sites via repeated weak antigen recognition, these outcomes may vary with other insulin-reactive T cells having different affinities. Given the complexity of T cell specificities and phenotypic variations in autoimmune diabetes,^76^ future studies using unbiased approaches to examine the entire autoimmune repertoire would be instructive.

## Concluding remarks

**Figure 1.**
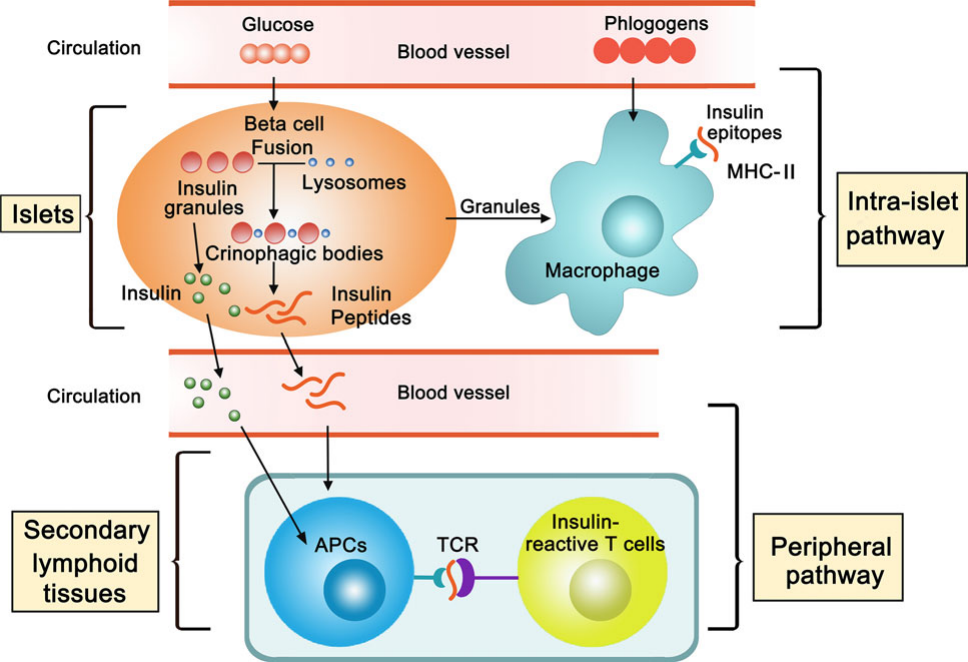
Two pathways of antigen presentation underlying initiation of type 1 diabetes. In the islet of Langerhans, the resident macrophages are in close contact with the β cells. This “β cell-macrophage synapse” involves extensive membrane activities and results in passage of β cell granules to the macrophages. In the context of autoimmunity, the macrophages that take up the granules adequately present β cell antigens to the T cells that have entered the islets (intra-islet pathway), a key process in mediation of local inflammation. The physiological roles of the macrophages at the steady state are less well understood. One possibility is to sense phlogogens in the blood and orchestrate the defense against pathogens. The β cells spontaneously generate insulin peptides in a set of vesicles termed crinophagic bodies. The crinophagic pathway is a natural mechanism by which the β cells catabolize excessive production of regular insulin dense core granules. In response to glucose, these catabolized products are released into circulation and can be presented by APCs in the secondary lymphoid tissues and recognized by T cells (peripheral pathway). Although the effects of the released peptides in T cell biology are not completely understood, one indicator T cell acquires an activation phenotype that promotes diabetogenicity. APC, antigen presenting cell ; MHC-II, major histocompatibility complex II; TCR, T cell receptor.

The propensity of tissue-specific autoimmunity is highly influenced by certain MHC haplotypes. It is long known that self-tolerance is established to lymphocytes recognizing native proteins, but that autoreactivity may occur when a denatured version of the same protein is available for recognition. Generation and release of catabolized protein fragments is a natural process of a target tissue; this finding may explain how tissue antigens can be presented to T cells *in situ* by resident APCs, and furthermore, it suggests a pathway by which self-peptides are made available in the lymphoid tissues where the lymphocytes can be sensitized (Fig. 1). From the perspective of autoimmunity, this process seems to be a “loophole” in a given individual that precipitates disease risks. However, it is expected that many peptides with undefined bioactivities may also be released. Recognition of such pMHCs may have evolutionary benefits that remain to be determined. We also suggest two therapeutic implications derived from the described studies. First, analyzing the peptidome released into the periphery may help to provide a targeted approach identifying antigens responsible for triggering the original T cell reactivity. Second, many antigen-specific strategies involve administration of self-peptides.^77–79^ Our findings regarding T cell biology suggest that caution is needed when administering self-peptides to achieve inducible tolerance. Considering the heterogeneity of T cell receptor affinities, the MHC densities, and the features of the APCs, administration of self-peptides, even in a tolerogenic manner, may mobilize pathogenic responses that counter the desired regulatory effects.
